# Predicting neurodevelopmental disorders using machine learning models and electronic health records – status of the field

**DOI:** 10.1186/s11689-024-09579-0

**Published:** 2024-11-15

**Authors:** Shyam Sundar Rajagopalan, Kristiina Tammimies

**Affiliations:** 1 Center of Neurodevelopmental Disorders (KIND), Centre for Psychiatry Research, Department of Women’s and Children’s Health, Karolinska Institutet and Child and Adolescent Psychiatry, Stockholm Health Care Services, Stockholm County Council, Stockholm, Sweden; 2 Institute of Bioinformatics and Applied Biotechnology, Bengaluru, India; 3 Astrid Lindgren Children’s Hospital, Karolinska University Hospital, Region Stockholm, Solna, Sweden

**Keywords:** Neurodevelopmental Disorder, Machine Learning, Electronic Health Record, Population Register

## Abstract

Machine learning (ML) is increasingly used to identify patterns that could predict neurodevelopmental disorders (NDDs), such as autism spectrum disorder (ASD) and attention-deficit hyperactivity disorder (ADHD). One key source of multilevel data for ML prediction models includes population-based registers and electronic health records. These can contain rich information on individual and familial medical histories and socio-demographics. This review summarizes studies published between 2010–2022 that used ML algorithms to develop predictive models for NDDs using population-based registers and electronic health records. A literature search identified 1191 articles, of which 32 were retained. Of these, 47% developed ASD prediction models and 25% ADHD models. Classical ML methods were used in 82% of studies and in particular tree-based prediction models performed well. The sensitivity of the models was lower than 75% for most studies, while the area under the curve (AUC) was greater than 75%. The most important predictors were patient and familial medical history and sociodemographic factors. Using private in-house datasets makes comparing and validating model generalizability across studies difficult. The ML model development and reporting guidelines were adopted only in a few recently reported studies. More work is needed to harness the power of data for detecting NDDs early.

**Supplementary Information:**

The online version contains supplementary material available at 10.1186/s11689-024-09579-0.

## Introduction

Neurodevelopmental disorders (NDDs) are childhood conditions impacting cognitive development, motor functioning, and higher-order executive functions, such as emotions, language, and memory. The Diagnostic and Statistical Manual of Mental Disorders (DSM-5) specifies that the symptoms of NDDs may be present in early development but not manifest fully into core diagnostic features till later in life [1]. NDDs are lifelong and encompass multiple conditions, including autism spectrum disorder (ASD), attention deficit hyperactivity disorder (ADHD), intellectual disability (ID), communication disorders, and motor disorders. NDDs frequently co-occur in individuals, and their deficit or excessive symptoms may overlap amongst conditions. Currently, there are no known biomarkers for any NDD [2, 3], and the diagnosis is given based on their phenotypic manifestations by trained clinicians observing an individual over time. The subjectivity in the diagnostic process and the lack of trained clinicians, especially in remote regions, leads to delays in diagnosis, depriving early intervention opportunities. Early diagnosis of NDDs can lead to early interventions, improved prognosis, and treatment outcomes [4].

Machine learning (ML) methods have the potential to manage the myriad of these complexities by developing robust prediction models for NDDs using various data [3]. If designed right, the power of prediction models comes from their generalization ability to predict unseen individual outcomes reliably across populations. The splurge in a massive amount of multimodal, high-dimensional healthcare data warrants advanced analytical approaches for mining them for subtle patterns, designing prediction models, and characterizing medical conditions. The learning process from the training data can be supervised or unsupervised, and the resulting learned information is captured in a *model*. The model thus developed is further applied for deriving insights into new unseen data and should generalize well across populations [5]. In the supervised learning process, the goal is to learn a mapping between the provided input–output label pairs, while in the unsupervised learning, in the absence of target labels, the aim is to identify hidden patterns in the data. The field of ML has made tremendous progress in the last two decades in natural language processing (NLP), computer vision (CV), and speech processing domains. In addition, ML models have shown promising results of predictive diagnostic abilities in cancer [6, 7], and cardiovascular diseases [8].

In the past few decades, an increasing number of nationwide population-based registers and digitized electronic health records (EHRs) have been administered to develop national-wide healthcare systems, social services, and research [9]. Such an ensemble of registers contains a large volume of rich information that can be exploited for developing robust and intelligent ML approaches to develop prediction, prognosis, and treatment response models for multiple health conditions [10–17] The EHRs contain information such as patient’s medical history, clinical visits, prescribed medicines, and sociodemographic details that may provide insights into the development of conditions.

ML models have shown great promise in screening and diagnostic prediction of NDDs using different modalities, such as clinical and behavioural assessments, genetics, and brain imaging techniques such as Electroencephalography (EEG) and Magnetic Resource Imaging (MRI) [3]. A common challenge with these modalities is the unavailability of labeled large sample sizes for building effective ML models. EHRs and population-based registers do not generally have this limitation and, therefore, could potentially have data to help construct NDD prediction models. However, the lack of easy access to EHR data stored in unstructured formats hinders the ability to perform analyses more efficiently. Despite this, studies utilizing ML methods for processing population-based registers and EHRs in the context of NDDs are emerging.

In this review, we sought to identify studies employing ML models with EHRs and population-based registers for predicting NDD. Our objectives included reporting the variables used for prediction and assessing model performance across studies. Additionally, we synthesized recommendations for future research based on our findings. We hypothesized that existing studies would exhibit heterogeneity in design, sample sizes, predictor variables, and outcome measurements. Such variability could challenge these models' comprehensive conclusions, generalizability, and clinical implementation. We provide a summary and evaluation of the current state of ML-based prediction models for NDD diagnosis, considering their quality and performance. We also address the challenges and potential avenues for the successful integration of ML in enhancing neurodevelopmental disorders diagnosis. To our knowledge, this is the first review focused on ML models leveraging EHRs and population-based registers for NDD prediction.

## Methods

### Protocol and information sources

We performed this scoping review according to the five-stage framework of Preferred Reporting Items for Systematic Reviews and Meta-Analyses Extension for Scoping Reviews (PRISMA) Checklist and Explanation [18–20]. We restricted the search to the articles to include peer-reviewed and published work between 2010 – 2022 followed with a narrow search for the first months of 2023. A literature search was performed in Medline, Embase, Cochrane Library, Web of Science and PsycInfo databases. A simplified search was also done in Google Scholar. An information specialist from Karolinska Institutet University library assisted in our initial literature search to screen articles for the final review.

### Search strategy and inclusion/exclusion criteria

The search strategy was developed in Medline (Ovid). For each search concept, the Medical Subject Headings (MeSH-terms) and free text terms were identified. The search was then translated into the other databases. No language restriction was applied. De-duplication was done using the method described by Bramer et al. [21].

Additionally, we compared DOIs of the identified articles as final step. After the initial searches, we performed a snowball search to check references and citations of eligible studies from the database searches using the tool—connectedpapers.com—and manually verifying the references section from selected articles in this study. A simplified search was also done in Google Scholar where the first 320 records were screened. The last search of articles was conducted 2022–09-27. To account for very recent published works, we conducted a simplified search for new articles published between Oct 2022—Mar 2023. The full search strategies for all databases are available in the Supplementary Material (Table S1—Table S5).

Our objective was to review articles that had developed prediction models for NDDs using ML methods and population-based registers, and EHRs. We included articles using classical ML and deep learning (DL) algorithms for model development. We excluded studies establishing associations between multiple factors and disease conditions. Also, we excluded grouping studies involving the identification of subcategories or comorbidities of NDDs.

### Selection of sources of evidence

The articles matching the inclusion criteria from the literature searches were exported into the Zotero citation tool. Thereafter, both authors sorted articles based on title, abstract, and inclusion criteria. The selected articles are further assessed by reviewing the full text of the article to match the inclusion criteria.

### Data extraction

To process the selected articles for information needed in the review, we first extracted a table of items from each publication and used them to summarize the study design and findings. These included data items, such as aim, sample size, predictive variables used in the study, method of handling unbalanced data, sparsity, heterogeneity, employed ML methods, their findings, limitations, performance variables and the used datasets.

## Results

**Fig. 1:**
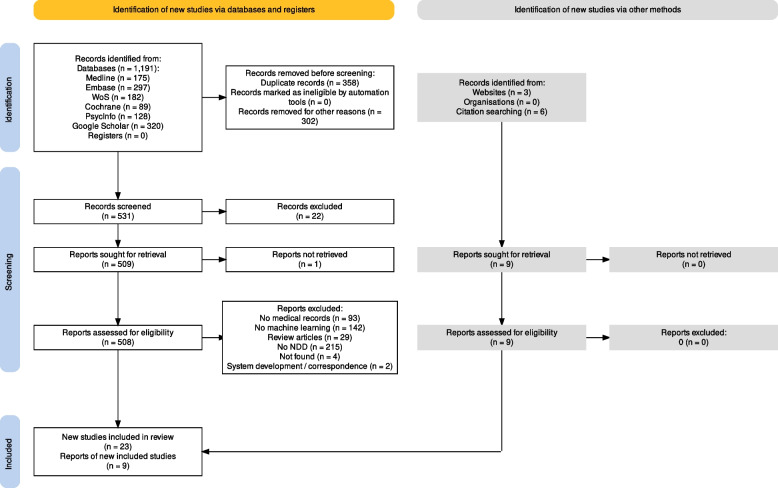
PRISMA flow diagram. After screening 1191 articles, 32 were retained for the review

**Fig. 2:**
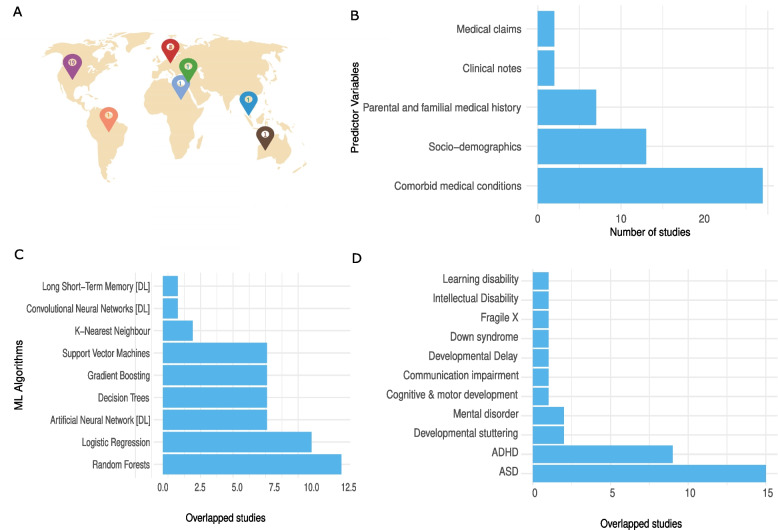
Distribution of reviewed articles across multiple factors. (**a**) number of articles from different countries, (**b**) usage of categories of predictor variables in studies, (**c**) ML algorithms used in studies, and (**d**) multiple co-occurring NDD conditions in studies

Our search strategy resulted in 1191 records, from which 302 were removed in the first step as these were meeting abstracts or proceedings and 358 as duplicate records (Fig. 1). After removing 683 from the initial list of 1191, we were left with 508. From this, we excluded records that did not use medical records (*n* = 93), did not use machine learning (*n* = 142), review articles (*n* = 29), no NDD (*n* = 215), records not found (*n* = 4), and system development/correspondence (*n* = 2). We further included 9 articles from other sources, 32 of which met the inclusion criteria and were included in this review, as summarized in the PRISMA flow diagram (Fig. 1). The included studies were conducted in 13 different countries. Most studies came from the USA (59%), followed by Denmark (6%). There is a single study each from the UK, Sweden, Germany, Finland, Switzerland, Netherlands, Egypt, Brazil, Israel, Thailand, and Australia (Fig. 2a, Table S6).

The sample sizes in the studies varied significantly between 50 participants and a dataset of 4.5 million subjects (Table 1). Prediction models were developed for a single diagnosis and a combination of NDDs. Approximately 47% of included studies developed ASD prediction models, 28% focused on ADHD models, and the remaining were individual studies for other NDDs (Fig. 2b, Table 1).

**Table 1:** Main characteristics of the reviewed articles

Author	Dataset	Variables	Sample Size	Method	Results
**ASD Prediction Models**					
Engelhard et al. [15]	EHR from Duke University Health System	Demographics, diagnosis and procedure codes, laboratory measurements, medications, vital signs, and encounter details	N: 45,080 ASD: 924	L2-regularized Cox proportional hazards (CoxPH), gradient-boosting survival analysis and random survival forest	Autism detection (360 days): Sensitivity: 59.8% PPV: 17.6% Specificity: 81.5%
Betts et al. [16]	Health Administrative Datasets from New South Wales, Australia	Clinical, demographic & lifestyle information and ICD10 AM (Australian version of the ICD-10) and Australian Classification of Health Intervention (ACHI) codes	N:261,447 mother baby dyads ASD: 981	Logistic regression with elastic net regularization and gradient boosting trees	AUC:0.73
Allesøe et al. [13]	Danish nationwide registers, family and patient diagnostic histories, birth-related measurements and genetics	Psychiatric diagnosis codes, age, parent diagnosis history, parent and patient infections, autoimmune diseases, diabetes, migraine, epilepsy	N: 63,535 ASD: 12,878 ADHD: 15,969 Controls: 20,681	Feed-forward DL network	Multidiagnostic prediction model: AUC: 0.8 MCC: 0.28
Onishchenko et al. [22]	**Truven** (claims), **UCM** (diagnostic records)	Comorbid disease categories	Truven ASD: 15,164 No ASD: 4,488,420 UCM ASD: 377 No ASD: 37,634	SLD, Long Short-Term Memory, Random Forest, Gradient Boosting	AUC: > 0.8
Bishop et al. [23]	EHR from the Marshfield Clinic	Age at death, sex, EHR length, 30 comorbidities, co-occurring ID and down syndrome	ASD: 91 No ASD: 6,186	Random Forest	Accuracy: 93% Sensitivity: 75% Specificity: 94% AUC: 0.88
Rahman et al. [24]	EMR from a Israeli Health Maintenance Organization	Socio demographics, parental medical histories, prescribed medications	ASD: 1, 397 No ASD: 94,741	Logistic Regression, Artificial Neural Network, Random Forest	Accuracy: 95.62% Sensitivity: 29.93% Specificity: 98.18% PPV: 43.35%
Hassan et al. [25]	National Database for Autism Research	Family and subject medical history	ASD: 2,577 Non-ASD: 410	Decision Tree	Accuracy: 89.2%
Maenner et al. [26]	Georgia site of the ADDM Network	Words and phrases contained in children’s developmental evaluations	ASD: 1,355 Non-ASD: 1,257	Random Forest	Sensitivity: 84%, PPV: 89.4% AUC: 0.932
Ejlskov et al. [27]	Danish Civil Registration System & Danish Medical Birth Register	Mental, cardiometabolic, neurologic, congenital defects, autoimmune, asthma, allergy conditions, birth weight, year, gestational age, parental age, education	ASD: 26,840 Non-ASD: 1,670,391	Random Forest, XGB, Generalized Linear Model, Elastic Net, Neural Networks, SVM, KNN, Ensemble Learning	AUC: > 0.6
Alexeeff et al. [28]	EMR and administrative claims for children in Northern California, Georgia, and the Northwest	79 medical conditions from 19 domains	ASD: 3,911 Non-ASD: 38,609	Clustering using Conditional Inference Tree	-
Lingren et al. [29]	EHR from the Boston children hospital, Cincinnati Children’s Hospital and Medical Center & Children’s Hospital of Philadelphia	ICD-9 codes and concepts from clinical notes	ASD: 20,658	Rules, SVM, Clustering	PPV: 0.786 Sensitivity: 0.769 F1: 0.761 AUC: 0.770
Leroy et al. [30]	EHRs frin the Arizona Developmental Disabilities Surveillance Program	Extraction of entities from text	N: 6,636 sentences	Rule based and Pruned Decision Tree	ML Precision: 60% Recall: 30%
Chen et al. [31]	Market Scan Health Claims Database 2005–2016	Disease CCS codes, sex, encounters of emergency department visits	ASD: 12,743 Non-ASD: 25,833	Logistic Regression, Random Forest	Sensitivity: 40% PPV: 20.5% Specificity: 96.4% AUC: 0.834
Yuan et al. [32]	Hand written semi-structured and unstructured medical forms of children	Lexical, lda & doc2vec features, parent and teacher, preschool and early intervention questionnaires, phone intake by social workers	ASD: 56 Non-ASD: 143	SVM	Accuracy: 83.4% Precision: 64.6% Recall: 91.1% F2: 84.2%
Lerthattasilp et al. [33]	Medical records from the Thammasat University Hospital	Gender, age, chief complaint, communication, birthweight, maternal & paternal age, family history of ASD or DD, caregiver education, history of the child’s ASD, and clinical observation symptoms	ASD: 104 Non- ASD: 35	Logistic Regression	AUC: 91%
**ADHD Prediction Models**					
GarciaArgibay et al. [17]	Population-based Swedish Registers	Psychiatric and somatic disorder ICD codes, sex, head circumference and weight at birth, small size for gestational age, Apgar score, number of failed subjects at school at age 16, and presence of criminal convictions	N: 238,696 ADHD: 12,893	Logistric regression, random forest, gradient boosting, XGBoost, DNN and ensemble models	AUC: 0.75
Shi et al [34]	Birth cohorts of children in Olmsted County of Minnesota	ICD-9 codes	ADHD: 237 LD: 162 No ADHD: 1,194	LASSO, Elastic Net Logistic Regression, Classification And Regression Trees, Stochastic Gradient Boosting	ADHD Accuracy: 0.96 Sensitivity: 0.69 Specificity: 0.99 PPV: 0.93
Mikolas et al. [35]	Medical records from Technical University Dresden	Age, gender, symptom ratings, Neuropsychological measures	ADHD: 153 No ADHD: 139	Linear SVM	Accuracy: 66.1% Sensitivity: 66.9% Specificity: 65.4% AUC: 0.66
Chen et al. [36]	Patient records from the South West Yorkshire Partnership NHS Foundation Trust	Demographics, screening questionaires and clinical interviews	N: 69	SVM, Logistic Regression, Naive Bayes, Random Forest, Decision Tree, KNN	Accuracy: 85.51% AUC: 0.871
Caye et al. [37]	Birth cohorts from ALSPAC, E-Risk, and Pelotas,The Multimodal Treatment Study of Children with ADHD (MTA)	Female sex, socio economic status, mother’s depression, IQ, maltreatment, ADHD, depressive symptoms, oppositional defiant behaviour & conduct disorders, and single parent	ALSPAC ADHD:5113 E-Risk ADHD:2040 Pelotas ADHD:4039 MTA ADHD:476 No ADHD:241	Logistic Regression, Random Forest, Stochastic Gradient Boosting, ANN	AUC: 0.82
Elujide et al. [38]	Medical records from Yaba Psychiatry Hospital, Yaba, Lagos State, Nigeria	Age, occupation, religion, spiritual consult, age code, faNoily, loss of parent, genetic, sex, status, injury & divorce	N: 500	DL, Multi Layer Perceptron, SVM, Random Forest, DecisionTree	Accuracy: 65% AUC: 0.73
Morris et al. [39]	Neurofibromatosis type 1 (NF1) clinical registry and EHR information from Washington University	Race, sex, family history of NF1, clinical features associated with NF1, and diagnosis codes	ADHD: 194 No ADHD: 384	Gradient Boosting	AUC: 0.74
Tran et al. [40]	The CEGS N-GRID 2016 Shared Task in Clinical NLP Records	Short text description of patient’s history of present illness	ADHD: 404 No ADHD: 582	SVM, CNN and ReHAN	micro-F: 63.144%
**Other NDDs Prediction Models**					
Movaghar et al. [41] (Fragile X syndrome)	EHR from Marshfield Clinic health-care system	ICD-9 codes	FXS: 55 No FXS: 5,500	Random Forest	AUC: 0.772
Koivu et al. [42] (Down syndrome)	Clinical records of two datasets from the Canada and one from the UK	Biological measurements, ethnicity, smoking, maternal age, patient weight, gestational age, NT, PAPP-A and fhCG measurements	Trisomy21:239 Controls:1611	SVM, Logistic Regression, Naive Bayes, Random Forest, Decision Tree, Deep Forward NN, KNN	AUC: 0.96
Gui et al. [43] (Neurodevelopment)	Medical records & MRI scans from University Hospitals of Geneva	Gestational Age, Birth Weight, Persistent ductus arteriosus, Birth asphyxia, presence of sepsis, Bronchopulmonary dysplasia, parental socioeconomic status	N: 84	Linear Discriminant Analysis	AUC: 0.77
Randolph et al. [44] (Neurodevelopment)	The NICHD NRN Generic Database (GDB)	Cord pH, BE, GA, BW, 5-min Apgar, SGA, multiple gestation, race/ethnicity, maternal insurance, hypertension, hemorrhage, antibiotics, ANS	N: 3,979 NDI/death: 2124	Logistic Regression	Accuracy: 70% Sensitivity: 0.71 Specificity: 0.64
Hill et al [45] (Commu-nication impairment)	Pediatric Health Information System from Children’s Hospital Association (Overland Park, KS)	Patient demographics, diagnoses, procedures, detailed pharmacy information	CI: 50, No CI: 86	Logistic Regression	Sensitivity: 82.6% Specificity: 86.3% AUC: 0.92
Pruett et al. [46] (Developmental stuttering)	Vanderbilt University Medical Center (VUMC) EHR	Child, adult onset fluency disorder, hearing loss, sleep disorders, atopy, codes for infections, neurological deficits, body weight	Stutter: 574 No Stutter: 2754	Decision Tree	PPV: > 83%
Shaw et al. [47] (Developmental stuttering)	Vanderbilt University’s EHR	Comorbid ICD-9 codes mapped to phecodes	Stutter: 141 No Stutter: 709	Classification And Regression Tree	PPV: 83% Sensitivity: 68.8%
Shrot et al. [48] (Intellectural development)	Edmond and Lily Safra Children’s Hospital, Sheba Medical Center EHR	Clinical and imaging data. Clinical data included genetic, demographic, and seizure characteristics	ID: 39 No ID: 38	Random Forest	Sensitivity: 0.69 Precision: 0.81 AUC: 0.68
van Dokkum et al. [49] (Developmental delay)	Community based cohort Longitudinal Preterm Outcome Project (LOLLIPOP)	Perinatal, parental factors and child growth milestones during the first two years	N: 1,983	Logistic Regression	Sensitivity: 73% Specificity: 80% AUC: 0.837

There were only three studies [13, 17, 27] that used Swedish and Danish nationwide population-based registers for the model development, and the rest of the studies used EHRs. Of the studies, 82% employed classical ML methods, while the remaining 18% applied DL methods (Fig. 2c, Table S7). The majority of the studies (60%) were published in the last three years.

The comorbid medical conditions, sociodemographics, and parent medical history were the most commonly used predictor variables (Fig. 2d, Table S8). In terms of performance results, the AUC metric was greater than 75% in most studies, while the sensitivity metric was less than 75% (Table 1, Table S9), indicating a need for further advancements. In the studies reviewed here, we did not find clear association between larger sample sizes and higher performance.

### Input preprocessing for addressing data quality issues

The data used in studies were from population-based registers, EHRs or medical records (diseases, medications, lab tests, procedures, clinical notes) of patients and family members, and insurance claim forms which all are characterized by missing values, the high dimensionality of records, heterogeneity, imbalance case–control categories, errors, and systematic biases. Data imputation is one of the commonly employed methods for handling missing data. In the reviewed studies, imputation was done in different ways, including using the random forest-based methods to impute the values [24], populating the missing data with the average value for continuous variables and mode value for discrete variables [36], replacing missing values either with zeros or unknown status values [42] and use chain equations from remaining predictors to fill missing values. Mikolas and colleagues filtered the features and participants with more than 20% of missing values [35], and Garcia-Argibay, et al. [17] included features with less than 10% missing values.

The case–control class/category imbalance problem was addressed using different tools, including downsampling the number of controls [27] or upsampling the number of cases [32] by randomly generating new samples between each positive sample and its nearest neighbors, employing the Synthetic Minority Over-sampling Technique (SMOTE) method to increase the cases [17, 24] or to assign more weights to cases while training a model [34].

There are no standard ways to process multimodal data from population-based registers and EHRs for generating an effective representation. Schuler et al. [50] proposed a generalized low-rank modeling framework to form efficient representations. This low-rank modeling framework was further used for downstream clustering applications. The advent and success of deep neural networks for efficient representation learning have led to the emergence of new studies to form efficient representations for EHRs data. Landi et al. [51] have proposed a representation learning model using word embeddings, convolutional neural networks, and autoencoders to transform patient history in EHRs into a set of low-dimensional vectors. They evaluated the generated representation for patient stratification across various conditions using clustering methods and demonstrated the effectiveness of the representation. Miotto et al. [52] have applied an unsupervised DL method using denoising autoencoders to generate a representation—*Deep Patient*—for each patient record in EHRs. They demonstrated the effectiveness of this representation by developing risk prediction models for various diseases for patients. Most studies included in this review have used custom methods to convert the input records into a multidimensional numerical vector, while some have grouped certain factors into features and used them as input to the model.

### Prediction features and ML methods

The predictor variables used in included studies were comorbid medical conditions from ICD-9 and ICD-10 codes, health problems, medical screening data, prescribed medications of a child, parental medical histories, medications, extended family history of mental and non-mental health conditions, socio-demographics, hospital admission/discharge, outpatient visit events, clinical notes and medical claims (Fig 2d, Table S8).

The predictor variables or features were processed multiple ways to generate a unique numerical representation for each subject before training an ML model. For example, Onishchenko et al. [22] developed digital biomarkers for ASD from past medical conditions. Autism comorbid risk score (ACoR) was estimated in the early years of a child with ASD comorbidities. The score was further conditioned on current screening scores to reduce the false positive rate. A diagnostic history model using time-series patient data across 17 disease categories was developed for each patient. Chen et al. [31] developed an ASD prediction model for young children at 18 mo, 24 mo, and 30 mo using medical claims data. They have examined all diagnosis and procedure codes of a child's medical encounters and used Clinical Classifications Software (CCS) software to form different disease categories. The total number of encounters for each CCS category, sex, and encounters of emergency department visits were used as predictor variables for the model. Ejlskov et al. [27] examined the feasibility of using extended family history of mental and non-mental conditions to predict the ASD risk. A large national Denmark cohort of medical history data of three generations of family members is used for developing ML models. Morbidity indicators across 73 disorders, including mental, cardiometabolic, neurologic, congenital defects, autoimmune, and asthma of family members, were used as predictor variables. Allesøe et al. [13] have employed a DL model for mental disorder prediction, including NDDs using nationwide register data, family and patient medical histories, birth-related measurements, and genetics.

Most studies (82%) trained one or more ASD prediction models using classical ML methods. The most commonly used methods were logistic regression and random forests. Onishchenko et al. [22] used Sequence Likelihood Defect (SLD) to measure the deviations in the observed time-series diagnostic events across positive and control cohorts. It was shown that this approach resulted in better performance than state-of-the-art ML algorithms. The proposed approach has few model parameters to learn, unlike conventional deep neural networks with a large set of parameters. Yuan et al. [32] developed an ASD prediction model from medical claim forms. The medical claim forms are preprocessed to extract textual content, and natural processing techniques were applied to derive text features. These features were used to build a Support Vector Machine (SVM) classifier for ASD prediction. There were nine studies utilizing DL methods. Tran et al. [40] investigated the feasibility of using a short textual description of clinical notes to predict the risk of future multiple mental conditions, including ADHD. The baseline model was developed using the SVM classifier and compared with deep network models, such as Convolutional Neural Networks (CNN) and Recurrent Neural Networks with Hierarchical Attention (ReHAN). The detailed list of ML algorithms used in included studies is presented in Table S7. Many studies have developed an ensemble of models using multiple ML algorithms.

The model evaluation methods used in studies involved k-fold cross-validation techniques and/or train-valid-test split methods. The performance of the algorithms was reported using the commonly used metrics, such as sensitivity, specificity, the area under the curve (AUC), positive predictive value (PPV), and accuracy. Half of the studies have reported AUC performance greater than 75%. While most studies have reported superior performance in some metrics, the sensitivity results were relatively low, as the majority showed a sensitivity of less than 75%. Not all studies have reported performance results across all evaluation metrics in a consistent way. The summary of performance evaluation metrics reported in studies is shown in Table 1, Table S9.

### ML model interpretability for feature importances and generalization

Model interpretability is key to healthcare problems as it helps identify influencing variables for decision-making needed by clinicians. It is worth mentioning that most of the studies in this review have addressed the interpretability aspects in sufficient detail. The findings from the included studies and the dominating predictor variables influencing model performance varied across studies. For example, for ASD models, Betts et al. [16] identified gender, maternal age at birth, delivery analgesia, maternal prenatal tobacco disorders, and low 5-min APGAR score as dominant risk factors for ASD. Rahman et al. [24] found that the features derived from predictor variables, such as parental age and parental medications, contributed to a better ML model performance. These predictors agree with prior studies. However, they noted that the performance metrics varied across different ML models, with no one clear model outperforming all metrics. Ejlskov et al. [27] found that the best-performing ML model—extreme gradient boosting (XGB)—identified indicators across mental conditions (ASD, ADHD, neurotic/stress disorders) and non-mental conditions (obesity, hypertension, and asthma) of family members. The study concluded that a comprehensive family history of mental and non-mental conditions could better predict ASD than considering only the immediate family history of ASD. Hassan et al. [25] aim was to identify etiological factors of ASD using subject and family medical histories. Among the 81 family history attributes, six of them—father anxiety, sibling PDD-NOS, father autism disorder, sibling learning disability, father development delay, and mother autism disorder—were highly predictive of ASD. The attributes from the subject medical history that were highly predictive of ASD were atypical language development, age at 3-word sentences, age of first words, disrupted sleep patterns, dietary, gastrointestinal problems, allergies, low birth weight, and ADHD. Gender comparisons highlighted unique and overlapping conditions. One of the significant findings from this study was that parental and sibling developmental delays were strongly associated with ASD. Chen et al. [31] found that for prediction at ages 24 months and 30 months, 30–40 predictor variables were sufficient to achieve stable prediction performance, whereas, for early prediction at 18 months, the model needed 50 predictor variables. For prediction at age 24 months, the identified important variables included sex, developmental and nervous system disorders, psychological and psychiatric services, respiratory system infections and symptoms, gastrointestinal-related diagnosis, ear and eye infections, perinatal conditions, and emergency department visits. Lerthattasilp et al. [33] developed a logistic regression-based ASD prediction model. The experiments identified delayed speech, a history of avoiding eye contact, a history of not showing objects to others, poor response when the clinician draws attention, and low frequency of social interaction as the influencing predictor variables.

Garcia-Argibay et al. [17] developed an ADHD prediction model from population-based Swedish national registers. They found that parents' criminal history, male sex, relative with ADHD, number of academic subjects failed, and speech/learning disabilities were the top features contributing to the model performance. Shi et al. [34] developed ADHD and LD prediction models. The main findings from the study were that complex ML models using ICD-9 codes perform well in ADHD identification. However, they did not offer significant differences compared to using a simple model with a single family of ICD9 codes for ADHD. For LD identification, the utility of clinical diagnostic codes was limited. Mikolas et al. [35] developed a predictive model to detect individuals with ADHD comorbid with psychiatric conditions in a population. The findings from the study were: (a) age, gender, and accuracy/reaction time were more critical than other features, and (b) The ADHD core symptoms reported by parents/teachers did not carry the degree of importance as commonly assumed. Instead, combining symptoms across different domains had strong predictive power for ADHD diagnosis. Elujide et al. [38] developed an ADHD prediction model and found that the factors influencing the model were sex, age, occupation, and marital status. van Dokkum et al. [49] developed a prediction model of development delay at age 4. The perinatal, parental, and child growth milestones of 1st two years were used as predictor variables. They found that sex, maternal educational level, pre-existing maternal obesity, smiling, speaking 2 to 3-word sentences, standing, and BMI z score at one year were features of high importance for the prediction model. Allesøe et al. [13] developed a cross-diagnostic mental disorder diagnosis prediction model and found that previous mental disorders and age were the most important predictors for multi-diagnostic prediction. In summary, the most common predictor categories of NDDs across studies were patient and familial medical history and sociodemographic factors. The specific predictor variables in these categories vary across studies, making it harder to draw more detailed conclusions.

Most studies have used cross-validation and train-validation-test techniques to report model performance. While these methods provide sufficient information about the model performance in a single dataset, model generalizability can be validated using multiple independent datasets across sites and populations. For example, Onishchenko et al. [22] have used data from the Truven dataset for training models and an independent UCM database for validation. Lingren et al. [29] have used cohorts from Boston Children's Hospital, Cincinnati Children's Hospital and Medical Center, The Children's Hospital of Philadelphia, and the Vanderbilt University Medical Center in their model validation. The ADHD prediction model developed by Caye et al. [37] was validated using three external birth cohorts. Koivu et al. [42] developed a Down Syndrome prediction model using datasets from Canada and the UK for training a model and validating the model using an independent dataset from Canada. In summary, studies utilizing cohorts from distinct populations and sites for model validation for generalizability are emerging. The results of individual studies are summarized in Table 1.

### Limitations in the included studies

While all studies reported superior performance of proposed individual models, there were not many performance comparisons across studies. There is some overlap of influencing predictive variables across studies; however, the variations in experimental conditions and target sample populations make it harder to form conclusive evidence. For instance, the sample set size varies from fifty participants to millions across studies. The risk of bias, either due to sex, gender, the proportion of cases vs. controls, target site, and populations, was not sufficiently discussed in most studies. More studies following standardized protocols and using common data are critical for reproducible research and moving toward the clinical utility of such models [53].

## Discussion

We reviewed the current status of the application of ML to develop prediction models for NDDs using population-based registers and EHRs. More studies started emerging in the last few years. More recently, Engelhard et al. [15] proposed an early autism prediction model from the EHRs data collected before age one year and showed its promise for integration with other screening tools. The predictive models of NDDs show promising results and open up possibilities for further detailed studies for model development and clinical adaptation. Open research questions and challenges need to be addressed before we see clinical translational value from such ML models.

Our analysis of the findings from the studies presented in this work reveals several key insights. The studies that achieved better performance metrics (AUC > 0.8) employed deep neural networks, classical tree based ML models and used subject-wise representation from past medical history for model training. This observation holds across prediction models for ASD, ADHD, and other neurodevelopmental disorders. For studies with limited dataset sizes and noticeable class imbalances, the strategy to balance dataset sizes between cases and controls using methods, such as SMOTE, resulted in improved performance [17, 24]. While only a few studies leveraged population-based registers, they consistently showcased commendable ML predictive performance, underscoring the value of these registers in predicting NDDs.

Many studies use simple regression models to derive associations between predictor variables and outcomes. Such classical statistical-based association studies often were limited by their ability to operate on a handful of predictor variables and challenges with dealing with non-linear relationships. The outcomes from association studies have less clinical translational value than efficient predictive models on large datasets with tens and thousands of predictor variables [54]. Prediction models using DL methods show great promise toward this goal [55]. One of the key initial steps in using deep neural networks working with high-dimensional, longitudinal population-based registers and EHRs is to efficiently learn a representation capturing the complex non-linear inter-relationships present in the data. There is good progress in successfully designing restricted Boltzmann machines, deep convolutional networks, recurrent neural networks, attention-based transformer networks, variational autoencoders, and deep feed-forward networks for effective representation learning [56–58]. These advancements can be effectively utilized for building better prediction models. We noticed from our review here, that the tree based classical machine learning models can also be a good substitute when large sample sizes are available.

While most studies reviewed in this work used curated private EHRs datasets, only a few studies leverage population-based registers; the private datasets prevent open and reproducible research, and the results reported are difficult to replicate due to a lack of dataset availability, limiting scientific progress. There are efforts to collect, curate, anonymize, and publicly share population-based registers and EHRs datasets to advance the research area [59–61]. In addition, standard protocols must be employed across cohorts, and a standard set of measures must be collected.

The methods, tools, and protocols used to collect population-based registers and EHRs vary significantly across sites and populations. Also, clinicians' subjectivity in assigning disease codes to individuals leads to more inconsistency of records across sites and populations [3, 13]. These problems result in large datasets having missing and inconsistent data for a specific set of individuals. To address these challenges, imputation techniques and representation learning methods are used to preprocess data before training ML models. It is necessary to have well-balanced datasets across cases and controls to train such models. This will not be the case with medical records, as the proportion of cases will be significantly less than controls. However, a significant limitation in studies utilizing EHR is the missingness of data and how it is imputed before developing the models. Missingness in EHR data can arise from various sources, including inconsistent data entry, differences in healthcare practices, and patient non-compliance. Addressing the reasons for missing data is crucial and it was not done in standard manner in the reviewed articles. If the reasons are not understood, an improper imputation can introduce biases and affect the validity of the ML models' predictions. Further work is needed in this space to bring consistency in overcoming these challenges. ML model development and reporting results using standard guidelines, such as TRIPOD [62], GREMLIN [63] and STROBE [64] are essential.

Specific to ML model development for healthcare, sample sizes, hyperparameter tuning, overfitting, model complexity, model interpretability, and generalization are key challenges to be addressed carefully [55]. Recently, de Hond et al. [53] reviewed the current guidelines and quality criteria used in artificial intelligence-based prediction models in healthcare and reported a lack of stricter guidelines for three phases of the predictive model pipeline. Based on our review, we note that not all studies have sufficiently discussed these training considerations. Also, the reporting of performance results varies across studies. Recently, Baker, et al. [65] observed similar varying results across studies in their systematic review on the applicability of machine learning in NDD prediction and understanding. One of the commonly used metrics to report the model performance is the AUC.

Translating these ML models into real-world implementations involves completing three steps [66]. Once the model has been successfully validated with in-house datasets, it needs to undergo external validations, i.e., testing the model performance across different sites, datasets, and populations for its generalizability, followed by implementation trials. Model interpretability is key to explaining the rationale for the model's decision to assist clinicians in making informed decisions. Currently, not many DL studies have focused on addressing the interpretability aspects of the model in the context of NDDs. In summary, more work is needed to implement ML models utilizing population-based registers and EHRs for NDDs. Future studies should focus on enabling public data availability for reproducible research, more standardization in data representation, experiment conditions, and performance reporting. The advancements in DL approaches and explainable AI should be explored for better performance and model interpretability.

## Conclusion

In conclusion, this review thoroughly analyzes prior studies leveraging population-based registers and EHRs for ML-driven prediction of NDDs. Our systematic exploration spanned data preprocessing, model selection, and evaluation stages. While most studies employed classical ML techniques, only a handful adopted deep learning. Nearly all delved into model interpretability, spotlighting key influencing factors. Notable predictive variables from registers and EHRs include patient and family medical history and sociodemographic details. However, model sensitivity generally lagged behind other metrics. Challenges like data sparsity, inconsistent disease coding, and heterogeneity often went unaddressed before model creation. Importantly, the majority of the data utilized was private.

A limited number of studies use these registers and EHRs for ML in NDDs, but these are emerging. Differing datasets and experimental setups hinder direct performance comparisons between them. Future work should prioritize data availability, standardize data representation and performance metrics, and explore advancements in deep learning and explainable AI for enhanced outcomes and interpretability.

## Supplementary Information

Supplementary Material 1.

Supplementary Material 2.

## Data Availability

All collected data from article is presented in the main and supplemental material.
