# Supplementary material for: Predicting neurodevelopmental disorders using machine learning models and electronic health records – status of the field
Source: J Neurodev Disord. 2024 Nov 15;16:63. doi: 10.1186/s11689-024-09579-0 (PMC11566279; doi:10.1186/s11689-024-09579-0)
Supplement: Supplementary file 1 — Supplementary Material 1. [file 11689_2024_9579_MOESM1_ESM.docx]

Springer Nature 2021 L^A^TEX template

Predicting neurodevelopmental disorders using machine learning models and electronic health records – status of the field: Supplementary Material

Shyam Sundar Rajagopalan^1,2*^ and Kristiina Tammimies^1,2*^

^1^Center of Neurodevelopmental Disorders (KIND), Centre for Psychiatry Research,

Department of Women’s and Children’s Health, Karolinska Institutet and Child and

Adolescent Psychiatry, Stockholm Health Care Services, Stockholm County Council, Stockholm, Sweden.

^2^Astrid Lindgren Children’s Hospital, Karolinska University Hospital, Region Stockholm, Solna, Sweden.

*Corresponding author(s). E-mail(s): Shyam.Rajagopalan@ki.se; Kristiina.Tammimies@ki.se;

1

**Table S1**: Medline

| Interface: Ovid MEDLINE(R) and Epub Ahead of Print, In-Process & Other Non-Indexed Citations and Daily  Date of Search: 27 September 2022  Number of hits: 175  Comment: In Ovid, two or more words are automatically searched as phrases; i.e. no quotation marks are needed | Field labels   - exp/ = exploded MeSH term - / = non exploded MeSH term - .ti,ab,kf. = title, abstract and author keywords - adjx = within x words, regardless of order - * = truncation of word for alternate endings |
| --- | --- |

Database(s): Ovid MEDLINE(R) and Epub Ahead of Print, In-Process, In-Data-Review & Other NonIndexed Citations and Daily 1946 to September 26, 2022 Search Strategy:

| **#** | **Searches** | **Results** |
| --- | --- | --- |
| 1 | exp Neurodevelopmental disorders/ | 203,843 |
| 2 | ((attention deficit or childhood disintegrative or childhood onset fluency or child behavio?r or communication or development* or intellectual* or language or learning or motor skill? or speech or sound or stereotypic movement or tic) adj3 (disorder? or disab*)).ti,ab,kf. | 111,348 |
| 3 | (adhd or asd or asperger* or autism* or autistic or dyscalculia* or dyslexia* or neurodevelopmental or tourette*).ti,ab,kf. | 136,286 |
| 4 | ((5p or bartholin* or beuren or brachmann-de lange or broad thumb* or chromosome 5 or cri du chat or coffin-siris or crying cat or cat cry or de barsy or de lange or down* or fifth digit or kleefstra or labhart* or patau* or prader* or royer* or rubinstein* or schinzel-giedion or willi* or fragile-x) adj2 syndrom*).ti,ab,kf. | 38,701 |
| 5 | or/1-4 | 334,446 |
| 6 | exp Artificial intelligence/ | 156,398 |
| 7 | (clustering or random forest or decision tree or ”area under the curve” or receiver operating characteristic* or natural language processing or neural network* or predict* model* or predict* algorithm? or support vector*).ti,ab,kf. | 402,562 |
| 8 | (artificial* adj3 intelligen*).ti,ab,kf. | 27,007 |
| 9 | ((deep or machine) adj3 learning).ti,ab,kf. | 101,540 |
| 10 | or/6-9 | 530,698 |
| 11 | exp Records/ | 295,474 |
| 12 | ((computerized or electronic* or health or medical or national or patient? or population?) adj3 (record* or registr* or register?)).ti,ab,kf. | 323,391 |
| 13 | (population based or medical encounter? or medical condition?).ti,ab,kf. | 187,686 |
| 14 | or/11-13 | 708,765 |
| 15 | 5 and 10 and 14 | 195 |
| 16 | limit 15 to yr=”2010 -Current” | 175 |

**Table S2**: Embase

| Interface: embase.com  Date of Search: 27 September 2022  Number of hits: 297  Comment: Emtree is the controlled vocabulary in Embase | | Field labels   - /exp = exploded Emtree term - /de = non exploded Emtree term - ti,ab,kw = title, abstract and author keywords - NEAR/x = within x words, regardless of order - * = truncation of word for alternate endings | | |
| --- | --- | --- | --- | --- |
| **#** | **Searches** | | **Results** |  |
| 1 | ’learning disorder’/de OR ’autism’/exp OR ’behavior disorder’/de OR ’attention deficit hyperactivity disorder’/de OR ’communication disorder’/exp OR ’psychomotor disorder’/exp OR ’mental deficiency’/exp OR ’intellectual impairment’/de | | 536,955 |  |
| 2 | ((’attention deficit’ OR ’childhood disintegrative’ OR ’childhood onset fluency’ OR ’child behavio$r’ OR communication OR development* OR intellectual* OR language OR learning OR ’motor skill$’ OR speech OR sound OR ’stereotypic movement’ OR tic) NEAR/3 (disorder$ OR disab*)):ti,ab,kw | | 149,516 |  |
| 3 | adhd:ti,ab,kw OR asd:ti,ab,kw OR asperger*:ti,ab,kw OR autism*:ti,ab,kw OR autistic:ti,ab,kw OR dyscalculia*:ti,ab,kw OR dyslexia*:ti,ab,kw OR neurodevelopmental:ti,ab,kw OR tourette*:ti,ab,kw | | 184,774 |  |
| 4 | ((5p OR bartholin* OR beuren OR ’brachmann-de lange’ OR ’broad thumb*’ OR  ’chromosome 5’ OR ’cri du chat’ OR ’coffin siris’ OR ’crying cat’ OR ’cat cry’ OR ’de barsy’ OR ’de lange’ OR down* OR ’fifth digit’ OR kleefstra OR labhart* OR patau* OR prader* OR royer* OR rubinstein* OR ’schinzel giedion’ OR willi* OR ’fragile x’) NEAR/2 syndrom*):ti,ab,kw | | 50,443 |  |
| 5 | #1 OR #2 OR #3 OR #4 | | 644,316 |  |
| 6 | ’ML’/exp OR ’artificial intelligence’/exp | | 356,865 |  |
| 7 | clustering:ti,ab,kw OR ’random forest’:ti,ab,kw OR ’decision tree’:ti,ab,kw OR ’area under the curve’:ti,ab,kw OR ’receiver operating characteristic*’:ti,ab,kw OR ’natural language processing’:ti,ab,kw OR ’neural network*’:ti,ab,kw OR ’predict* model*’:ti,ab,kw OR ’predict* algorithm$’:ti,ab,kw OR ’support vector*’:ti,ab,kw | | 510,100 |  |
| 8 | (artificial* NEAR/3 intelligen*):ti,ab,kw | | 31,985 |  |
| 9 | ((deep OR machine) NEAR/3 learning):ti,ab,kw | | 118,448 |  |
| 10 | #6 OR #7 OR #8 OR #9 | | 754,334 |  |
| 11 | ’medical record’/exp OR ’register’/exp | | 481,611 |  |
| 12 | ((computerized OR electronic* OR health OR medical OR national OR patient$  OR population$) NEAR/3 (record* OR registr* OR register$)):ti,ab,kw | | 544,007 |  |
| 13 | ’population based’:ti,ab,kw OR ’medical encounter$’:ti,ab,kw OR ’medical condition$’:ti,ab,kw | | 255,511 |  |
| 14 | #11 OR #12 OR #13 | | 994,940 |  |
| 15 | #5 AND #10 AND #14 | | 464 |  |
| 16 | #5 AND #10 AND #14 AND ([article]/lim OR [article in press]/lim OR  [review]/lim) | | 320 |  |
| 17 | #5 AND #10 AND #14 AND ([article]/lim OR [article in press]/lim OR  [review]/lim) AND [2010-2022]/py | | 297 |  |

**Table S3**: Cochrane Library

| Interface: Wiley  Date of Search: 27 September  2022  Number of hits: 89 | | Field labels   - ti,ab,kw = title, abstract and author keywords - NEAR/x = within x words, regardless of order - * = truncation of word for alternate endings | | |
| --- | --- | --- | --- | --- |
| **#** | **Searches** | | **Results** |  |
| 1 | ((”attention deficit”:ti,ab,kw OR ”childhood disintegrative”:ti,ab,kw OR ”childhood onset fluency”:ti,ab,kw OR (”child” NEXT behavio?r):ti,ab,kw OR communication:ti,ab,kw OR development*:ti,ab,kw OR intellectual*:ti,ab,kw OR language:ti,ab,kw OR learning:ti,ab,kw OR (”motor” NEXT skill?):ti,ab,kw OR speech:ti,ab,kw OR sound:ti,ab,kw OR ”stereotypic movement”:ti,ab,kw OR  tic:ti,ab,kw) NEAR/3 (disorder?:ti,ab,kw OR disab*:ti,ab,kw)) | | 14,377 |  |
| 2 | (adhd:ti,ab,kw OR asd:ti,ab,kw OR asperger*:ti,ab,kw OR autism*:ti,ab,kw OR autistic:ti,ab,kw OR dyscalculia*:ti,ab,kw OR dyslexia*:ti,ab,kw OR neurodevelopmental:ti,ab,kw OR tourette*:ti,ab,kw) | | 13,575 |  |
| 3 | ((5p:ti,ab,kw OR bartholin*:ti,ab,kw OR beuren:ti,ab,kw OR ”brachmann-de lange”:ti,ab,kw OR (”broad” NEXT thumb*):ti,ab,kw OR ”chromosome 5”:ti,ab,kw OR ”cri du chat”:ti,ab,kw OR coffin-siris:ti,ab,kw OR ”crying cat”:ti,ab,kw OR ”cat cry”:ti,ab,kw OR ”de barsy”:ti,ab,kw OR ”de lange”:ti,ab,kw OR down*:ti,ab,kw OR ”fifth digit”:ti,ab,kw OR kleefstra:ti,ab,kw OR labhart*:ti,ab,kw OR patau*:ti,ab,kw OR prader*:ti,ab,kw OR royer*:ti,ab,kw OR rubinstein*:ti,ab,kw OR schinzel-giedion:ti,ab,kw OR willi*:ti,ab,kw OR fragile-x:ti,ab,kw) NEAR/2 syndrom*:ti,ab,kw) | | 56,882 |  |
| 4 | #1 OR #2 OR #3 | | 77,178 |  |
| 5 | (clustering:ti,ab,kw OR ”random forest”:ti,ab,kw OR ”decision tree”:ti,ab,kw OR  ”area under the curve”:ti,ab,kw OR (”receiver operating” NEXT  characteristic*):ti,ab,kw OR ”natural language processing”:ti,ab,kw OR (”neural”  NEXT network*):ti,ab,kw OR (predict* NEXT model*):ti,ab,kw OR (predict*  NEXT algorithm?):ti,ab,kw OR (”support” NEXT vector*):ti,ab,kw) | | 30,857 |  |
| 6 | (artificial*:ti,ab,kw NEAR/3 intelligen*:ti,ab,kw) | | 1,185 |  |
| 7 | ((deep:ti,ab,kw OR machine:ti,ab,kw) NEAR/3 learning:ti,ab,kw) | | 2,575 |  |
| 8 | #5 OR #6 OR #7 | | 32,690 |  |
| 9 | ((computerized:ti,ab,kw OR electronic*:ti,ab,kw OR health:ti,ab,kw OR medical:ti,ab,kw OR national:ti,ab,kw OR patient?:ti,ab,kw OR population?:ti,ab,kw) NEAR/3 (record*:ti,ab,kw OR registr*:ti,ab,kw OR register?:ti,ab,kw)) | | 37,421 |  |
| 10 | (”population based”:ti,ab,kw OR (”medical” NEXT encounter?):ti,ab,kw OR  (”medical” NEXT condition?):ti,ab,kw) | | 9,917 |  |
| 11 | #9 OR #10 | | 46,385 |  |
| 12 | #4 AND #8 AND #11 with Publication Year from 2010 to 2022, with Cochrane  Library publication date Between Jan 2010 and Dec 2022, in Trials | | 89 |  |

**Table S4**: Web of Science Core Collection

| Interface: Clarivate Analytics  Editions = A&HCI , ESCI ,  SCI-EXPANDED , SSCI  Date of Search: 27 September  2022  Number of hits: 182 | | Field labels   - TS/Topic = title, abstract, author keywords and Keywords Plus - NEAR/x = within x words, regardless of order - * = truncation of word for alternate endings   Note: the Exact search-function was used for all the searches | | |
| --- | --- | --- | --- | --- |
| **#** | **Searches** | | **Results** |  |
| 1 | TS=((”attention deficit” OR ”childhood disintegrative” OR ”childhood onset fluency” OR ”child behavio$r” OR communication OR development* OR  intellectual* OR language OR learning OR ”motor skill$” OR speech OR sound OR  ”stereotypic movement” OR tic) NEAR/3 (disorder$ OR disab*)) | | 161,270 |  |
| 2 | TS=(adhd OR asd OR asperger* OR autism* OR autistic OR dyscalculia* OR dyslexia* OR neurodevelopmental OR tourette*) | | 187,253 |  |
| 3 | TS=((5p OR bartholin* OR beuren OR ”brachmann-de lange” OR ”broad thumb*” OR ”chromosome 5” OR ”cri du chat” OR coffin-siris OR ”crying cat” OR ”cat cry” OR ”de barsy” OR ”de lange” OR down* OR ”fifth digit” OR kleefstra OR labhart* OR patau* OR prader* OR royer* OR rubinstein* OR schinzel-giedion OR willi* OR fragile-x) NEAR/2 syndrom*) | | 53,409 |  |
| 4 | #3 OR #2 OR #1 | | 332,851 |  |
| 5 | TS=(clustering OR ”random forest” OR ”decision tree” OR ”area under the curve”  OR ”receiver operating characteristic*” OR ”natural language processing” OR ”neural network*” OR ”predict* model*” OR ”predict* algorithm$” OR ”support vector*”) | | 870,374 |  |
| 6 | TS=(artificial* NEAR/3 intelligen*) | | 77,435 |  |
| 7 | TS=((deep OR machine) NEAR/3 learning) | | 268,150 |  |
| 8 | #7 OR #6 OR #5 | | 1,033,781 |  |
| 9 | TS=((computerized OR electronic* OR health OR medical OR national OR patient$ OR population$) NEAR/3 (record* OR registr* OR register$)) | | 322,217 |  |
| 10 | TS=(”population based” OR ”medical encounter$” OR ”medical condition$”) | | 204,016 |  |
| 11 | #10 OR #9 | | 504,374 |  |
| 12 | #11 AND #8 AND #4 | | 197 |  |
| 13 | #11 AND #8 AND #4 and 2010 or 2012 or 2013 or 2014 or 2022 or 2021 or 2020 or  2019 or 2018 or 2017 or 2016 or 2015 (Publication Years) | | 182 |  |

**Table S5**: Psycinfo

| Interface: EBSCO  Date of Search: 27 September 2022  Number of hits: 128 | | Field labels   - DE = subject heading - TI = title - AB = abstract - KW = author keywords - Nx = within x words, regardless of order - * = truncation of word for alternate endings | | |
| --- | --- | --- | --- | --- |
| **#** | **Searches** | | **Results** |  |
| S1 | DE ”Neurodevelopmental Disorders” OR DE ”Attention Deficit Disorder” OR DE ”Attention Deficit Disorder with Hyperactivity” OR DE ”Autism Spectrum  Disorders” OR DE ”Autistic Traits” OR DE ”Developmental Disabilities” OR DE  ”Specific Language Impairment” OR DE ”Disruptive Behavior Disorders” OR DE ”Conduct Disorder” OR DE ”Oppositional Defiant Disorder” OR DE ”Emotional and Behavioral Disorders” OR DE ”Intellectual Development Disorder” OR DE  ”Anencephaly” OR DE ”Crying Cat Syndrome” OR DE ”Down’s Syndrome” OR  DE ”Tay Sachs Disease” OR DE ”Learning Disorders” OR DE ”Learning Disabilities” OR DE ”Reading Disabilities” | | 179,865 |  |
| S2 | TI ( ((”attention deficit” OR ”childhood disintegrative” OR ”childhood onset fluency” OR ”child behavio#r” OR communication OR development* OR intellectual* OR language OR learning OR ”motor skill#” OR speech OR sound OR ”stereotypic movement” OR tic) N3 (disorder# OR disab*)) ) OR AB (  ((”attention deficit” OR ”childhood disintegrative” OR ”childhood onset fluency” OR ”child behavio#r” OR communication OR development* OR intellectual* OR language OR learning OR ”motor skill#” OR speech OR sound OR ”stereotypic movement” OR tic) N3 (disorder# OR disab*)) ) OR KW ( ((”attention deficit” OR ”childhood disintegrative” OR ”childhood onset fluency” OR ”child behavio#r”  OR communication OR development* OR intellectual* OR language OR learning  OR ”motor skill#” OR speech OR sound OR ”stereotypic movement” OR tic) N3 (disorder# OR disab*)) ) | | 115,180 |  |
| S3 | TI ( (adhd OR asd OR asperger* OR autism* OR autistic OR dyscalculia* OR dyslexia* OR neurodevelopmental OR tourette*) ) OR AB ( (adhd OR asd OR asperger* OR autism* OR autistic OR dyscalculia* OR dyslexia* OR neurodevelopmental OR tourette*) ) OR KW ( (adhd OR asd OR asperger* OR autism* OR autistic OR dyscalculia* OR dyslexia* OR neurodevelopmental OR tourette*) ) | | 114,234 |  |

| S4 | TI ( ((5p OR bartholin* OR beuren OR ”brachmann-de lange” OR ”broad thumb*” OR ”chromosome 5” OR ”cri du chat” OR coffin-siris OR ”crying cat” OR ”cat cry” OR ”de barsy” OR ”de lange” OR down* OR ”fifth digit” OR kleefstra OR labhart* OR patau* OR prader* OR royer* OR rubinstein* OR schinzel-giedion OR willi* OR fragile-x) N2 syndrom*) ) OR AB ( ((5p OR bartholin* OR beuren OR ”brachmann-de lange” OR ”broad thumb*” OR ”chromosome 5” OR ”cri du chat” OR coffin-siris OR ”crying cat” OR ”cat cry” OR ”de barsy” OR ”de lange” OR down* OR ”fifth digit” OR kleefstra OR labhart* OR patau* OR prader* OR royer* OR rubinstein* OR schinzel-giedion OR willi* OR fragile-x) N2 syndrom*) ) OR KW ( ((5p OR bartholin* OR beuren OR ”brachmann-de lange” OR ”broad thumb*” OR ”chromosome 5” OR ”cri du chat” OR coffin-siris OR ”crying cat” OR ”cat cry” OR ”de barsy” OR ”de lange” OR down* OR ”fifth digit” OR kleefstra  OR labhart* OR patau* OR prader* OR royer* OR rubinstein* OR schinzel-giedion OR willi* OR fragile-x) N2 syndrom*) ) | 12,308 |
| --- | --- | --- |
| S5 | S1 OR S2 OR S3 OR S4 | 245,968 |
| S6 | ((DE ”ML” OR DE ”Artificial Intelligence” OR DE ”Computational Reinforcement  Learning” OR DE ”Inductive Logic Programming” OR DE ”ML Algorithms” OR  DE ”Extreme Learning Machine” OR DE ”Unsupervised Learning” OR DE  ”Pattern Recognition (Computer Science)” OR DE ”Feature Extraction” OR DE  ”Image Analysis”) AND (DE ”Affective Computing” OR DE ”Cognitive  Computing” OR DE ”Knowledge Engineering” OR DE ”Knowledge Representation”  OR DE ”Semantic Networks” OR DE ”Natural Language Processing”)) OR (DE  ”Artificial Neural Networks” OR DE ”Deep Neural Networks”) | 5,854 |
| S7 | TI ( (clustering OR ”random forest” OR ”decision tree” OR ”area under the curve”  OR ”receiver operating characteristic*” OR ”natural language processing” OR ”neural network*” OR ”predict* model*” OR ”predict* algorithm#” OR ”support vector*”) ) OR AB ( (clustering OR ”random forest” OR ”decision tree” OR ”area under the curve” OR ”receiver operating characteristic*” OR ”natural language processing” OR ”neural network*” OR ”predict* model*” OR ”predict* algorithm#” OR ”support vector*”) ) OR KW ( (clustering OR ”random forest” OR ”decision tree” OR ”area under the curve” OR ”receiver operating characteristic*” OR ”natural language processing” OR ”neural network*” OR  ”predict* model*” OR ”predict* algorithm#” OR ”support vector*”) ) | 55,682 |
| S8 | TI (artificial* N3 intelligen*) OR AB (artificial* N3 intelligen*) OR KW (artificial*  N3 intelligen*) | 7,345 |
| S9 | TI ( ((deep OR machine) N3 learning) ) OR AB ( ((deep OR machine) N3 learning)  ) OR KW ( ((deep OR machine) N3 learning) ) | 14,047 |
| S10 | S6 OR S7 OR S8 OR S9 | 70,424 |
| S11 | DE ”Medical Records” OR DE ”Client Records” OR DE ”Electronic Health Records” | 7,572 |
| S12 | TI ( ((computerized OR electronic* OR health OR medical OR national OR patient# OR population#) N3 (record* OR registr* OR register#)) ) OR AB ( ((computerized OR electronic* OR health OR medical OR national OR patient# OR population#) N3 (record* OR registr* OR register#)) ) OR KW (  ((computerized OR electronic* OR health OR medical OR national OR patient#  OR population#) N3 (record* OR registr* OR register#)) ) | 152,921 |
| S13 | TI ( (”population based” OR ”medical encounter#” OR ”medical condition#”) ) OR AB ( (”population based” OR ”medical encounter#” OR ”medical condition#”) ) OR KW ( (”population based” OR ”medical encounter#” OR ”medical condition#”) ) | 33,972 |

8 *Supplementary Material*

| S14 | S11 OR S12 OR S13 | 184,684 |
| --- | --- | --- |
| S15 | S5 AND S10 AND S14 | 145 |
| S16 | S5 AND S10 AND S14 Limiters - Publication Year: 2010-2022, Academic journals | 128 |

**Table S6**: Study countries **Table S7**: ML algorithms employed in studies

| \| **Country** \| **No of**  **studies** \| \| --- \| --- \| \| USA \| 19 \| \| Denmark \| 2 \| \| Sweden \| 1 \| \| Germany \| 1 \| \| Finland \| 1 \| \| Switzerland \| 1 \| \| Netherlands \| 1 \| \| UK \| 1 \| \| Egypt \| 1 \| \| Thailand \| 1 \| \| Australia \| 1 \| \| Brazil \| 1 \| \| Israel \| 1 \| | \| Algorithm \| Studies \| Count \| \| --- \| --- \| --- \| \| Generalized Linear  Models \| [1] \| 1 \| \| Logistic Regression \| [2], [3],  [4], [5],  [6], [7],  [8], [9],  [10], [11] \| 10 \| \| K-Nearest Neighbour  (kNN) \| [1], [7] \| 2 \| \| Support Vector Machine  (SVM) \| [1], [12],  [13], [14],  [7], [15],  [16] \| 7 \| \| Decision Tree \| [17], [18], [19], [7],  [15], [20],  [21] \| 7 \| \| Random Forests \| [22], [3],  [23], [4],  [1], [24],  [25], [26],  [7], [8],  [15], [27] \| 12 \| \| Gradient Boosting \| [22], [2],  [3], [1],  [6], [8],  [28] \| 7 \| \| Artificial Neural Network \| [3], [4],  [1], [7],  [8], [15],  [29] \| 7 \| \| Convolutional Neural  Network (CNN) \| [16] \| 1 \| \| Long Short-Term Memory (LSTM) \| [14] \| 1 \| \| Clustering \| [30], [12] \| 2 \| \| Others \| [22], [31],  [6], [7],  [32] \| 5 \| |
| --- | --- | --- | --- | --- | --- | --- | --- | --- | --- | --- | --- | --- | --- | --- | --- | --- | --- | --- | --- | --- | --- | --- | --- | --- | --- | --- | --- | --- | --- | --- | --- | --- | --- | --- | --- | --- | --- | --- | --- | --- | --- | --- | --- | --- | --- | --- | --- | --- | --- | --- | --- | --- | --- | --- | --- | --- | --- | --- | --- | --- | --- | --- | --- | --- | --- | --- | --- | --- |

| Metrics | 0% - 50% | 51% - 75% | 76% - 100% |
| --- | --- | --- | --- |
| Accuracy | -  **(0)** | [14], [10],  [15]  **(3)** | [23], [4], [6],  [19], [17]  **(5)** |
| Sensitivity | [4], [25]  **(2)** | [22], [23], [6],  [14], [10],  [27], [11]  **(7)** | [9], [12], [24]  **(3)** |
| Specificity | -  **(0)** | [14], [10]  **(2)** | [23], [4], [25],  [6], [9], [11]  **(6)** |
| AUC | -  **(0)** | [2], [3], [1],  [14], [15],  [28], [27]  **(7)** | [26], [31],  [23], [12],  [24], [25], [5],  [13], [19], [7],  [8], [32], [9],  [11], [29]  **(15)** |

| Variable | Studies | Count |
| --- | --- | --- |
| Comorbid medical conditions from ICD-9, ICD-10 codes, health problems, medical screening data, prescribed medications of a child | [22], [2], [3],  [31], [23],  [17], [30],  [12], [5], [25],  [24], [6], [26],  [14], [19], [7],  [8], [15], [32],  [9], [28], [10],  [21], [27],  [16], [11],  [29] | 27 |
| Parental medical histories, medications | [4], [17], [7],  [32], [10],  [11], [29] | 7 |
| Extended family history of mental and non-mental health conditions | [1] | 1 |
| Sociodemographics | [2], [22], [3],  [33], [5], [14],  [19], [7], [8],  [15], [28],  [10], [27] | 13 |
| Hospital admission/discharge, outpatient visit events | - | 0 |
| Clinical notes | [12], [24] | 2 |
| Medical claims | [25], [13] | 2 |

**Table S8**: Predictor variables used for model **Table S9**: Performance metrics reported in studies development

# References

1. Ejlskov, L. *et al.* Prediction of Autism Risk From Family Medical History Data Using Machine Learning: A National Cohort Study From Denmark. *Biological Psychiatry Global Open Science* **1** (2), 156–164 (2021). URL [https://www.sciencedirect.com/science/ article/pii/S2667174321000136.](https://www.sciencedirect.com/science/article/pii/S2667174321000136) [https: //doi.org/10.1016/j.bpsgos.2021.04.007](https://doi.org/10.1016/j.bpsgos.2021.04.007) .
2. Betts, K. S., Chai, K., Kisely, S. & Alati, R. Development and validation of a machine learning-based tool to predict autism among children. *Autism Research* **n/a** (n/a) (2023). URL [https://onlinelibrary. wiley.com/doi/abs/10.1002/aur.2912.](https://onlinelibrary.wiley.com/doi/abs/10.1002/aur.2912) [https://doi.org/10.1002/aur.2912,](https://doi.org/10.1002/aur.2912) eprint:

*Pediatrics* **20** (1), 510 (2020). URL [https:// doi.org/10.1186/s12887-020-02411-3.](https://doi.org/10.1186/s12887-020-02411-3) [https: //doi.org/10.1186/s12887-020-02411-3](https://doi.org/10.1186/s12887-020-02411-3) .

1. Koivu, A., Korpim¨aki, T., Kivel¨a, P., Pahikkala, T. & Sairanen, M. Evaluation of machine learning algorithms for improved risk assessment for Down’s syndrome. *Computers in Biology and Medicine* **98**, 1–7 (2018). URL [https://linkinghub.elsevier. com/retrieve/pii/S0010482518301112.](https://linkinghub.elsevier.com/retrieve/pii/S0010482518301112) [https://doi.org/10.1016/j.compbiomed.2018.](https://doi.org/10.1016/j.compbiomed.2018.05.004)

[05.004](https://doi.org/10.1016/j.compbiomed.2018.05.004) .

| https://onlinelibrary.wiley.com/doi/pdf/10.1002/aur.2912ple. *Epidemiology and Psychiatric Sciences* |
| --- |

1. Caye, A. *et al.* A risk calculator to predict adult attention-deficit/hyperactivity disorder: generation and external validation in three birth cohorts and one clinical sam-

.

1. Garcia-Argibay, M. *et al.* Predicting childhood and adolescent attentiondeficit/hyperactivity disorder onset: a nationwide deep learning approach. *Molecular Psychiatry* **28** (3), 1232– 1239 (2023). URL [https://www.nature. com/articles/s41380-022-01918-8.](https://www.nature.com/articles/s41380-022-01918-8) [https:](https://doi.org/10.1038/s41380-022-01918-8)

[//doi.org/10.1038/s41380-022-01918-8,](https://doi.org/10.1038/s41380-022-01918-8) number: 3 Publisher: Nature Publishing Group .

1. Rahman, R. *et al.* Identification of newborns at risk for autism using electronic medical records and machine learning. *European Psychiatry: The Journal of the Association of European Psychiatrists* **63** (1), e22 (2020). <https://doi.org/10.1192/j.eurpsy.2020.17> .
2. Lerthattasilp, T., Tanprasertkul, C. & Chunsuwan, I. Development of clinical prediction rule for diagnosis of autistic spectrum disorder in children. *Mental Illness* **12** (1), 7–16 (2020). URL [https://ovidsp.ovid.com/ ovidweb.cgi?T=JS&CSC=Y&NEWS=N& PAGE=fulltext&D=pmnm&AN=32742626.](https://ovidsp.ovid.com/ovidweb.cgi?T=JS&CSC=Y&NEWS=N&PAGE=fulltext&D=pmnm&AN=32742626) <https://doi.org/10.1108/MIJ-01-2020-0001> .
3. Shi, Y. *et al.* Utility of medical record diagnostic codes to ascertain attentiondeficit/hyperactivity disorder and learning disabilities in populations of children. *BMC*

**29**, e37 (2019). [https://doi.org/10.1017/ S2045796019000283](https://doi.org/10.1017/S2045796019000283) .

1. Hill, D. L. *et al.* Identifying CommunicationImpaired Pediatric Patients Using Detailed Hospital Administrative Data. *Hospital Pediatrics* **6** (8), 456–467 (2016). URL [https://ovidsp.ovid.com/ovidweb. cgi?T=JS&CSC=Y&NEWS=N&PAGE= fulltext&D=med13&AN=27381628.](https://ovidsp.ovid.com/ovidweb.cgi?T=JS&CSC=Y&NEWS=N&PAGE=fulltext&D=med13&AN=27381628)

<https://doi.org/10.1542/hpeds.2015-0154> .

1. Randolph, D. A. *et al.* Outcomes of Extremely Low Birth Weight Infants with Acidosis at Birth. *Archives of disease in childhood. Fetal and neonatal edition* **99** (4), F263–F268 (2014). URL [https://www.ncbi.nlm.nih.gov/ pmc/articles/PMC4274605/.](https://www.ncbi.nlm.nih.gov/pmc/articles/PMC4274605/) [https:](https://doi.org/10.1136/archdischild-2013-304179)

[//doi.org/10.1136/archdischild-2013-304179](https://doi.org/10.1136/archdischild-2013-304179)

.

1. van Dokkum, N. H., Reijneveld, S. A., Heymans, M. W., Bos, A. F. & de Kroon, M. L. A. Development of a Prediction Model to Identify Children at Risk of Future Developmental Delay at Age 4 in a Population-Based Setting. *International Journal of Environmental Research and Public Health* **17** (22), 8341 (2020). URL [https://www.ncbi.nlm. nih.gov/pmc/articles/PMC7698029/.](https://www.ncbi.nlm.nih.gov/pmc/articles/PMC7698029/) [https: //doi.org/10.3390/ijerph17228341](https://doi.org/10.3390/ijerph17228341) .
2. Lingren, T. *et al.* Electronic Health Record Based Algorithm to Identify Patients with Autism Spectrum Disorder. *PloS One* **11** (7), e0159621 (2016). [https://doi.org/10.1371/ journal.pone.0159621](https://doi.org/10.1371/journal.pone.0159621) .
3. Yuan, J., Holtz, C., Smith, T. & Luo, J. Autism spectrum disorder detection from semistructured and unstructured medical data. *EURASIP Journal on Bioinformatics and Systems Biology* **2017**, 3 (2017). URL [https://www.ncbi.nlm.nih.gov/ pmc/articles/PMC5288414/.](https://www.ncbi.nlm.nih.gov/pmc/articles/PMC5288414/) [https://doi. org/10.1186/s13637-017-0057-1](https://doi.org/10.1186/s13637-017-0057-1) .
4. Mikolas, P. *et al.* Training a machine learning classifier to identify ADHD based on real-world clinical data from medical records. *Scientific Reports* **12** (1), 12934 (2022). [https: //doi.org/10.1038/s41598-022-17126-x](https://doi.org/10.1038/s41598-022-17126-x) .
5. Elujide, I. *et al.* Application of deep and machine learning techniques for multi-label classification performance on psychotic disorder diseases. *Informatics in Medicine Unlocked* **23** (2021). URL [https://www.embase.com/ search/results?subaction=viewrecord& id=L2011532322&from=exporthttp: //dx.doi.org/10.1016/j.imu.2021.100545.](https://www.embase.com/search/results?subaction=viewrecord&id=L2011532322&from=export%20http://dx.doi.org/10.1016/j.imu.2021.100545) <https://doi.org/10.1016/j.imu.2021.100545> .
6. Tran, T. & Kavuluru, R. Predicting mental conditions based on “history of present illness” in psychiatric notes with deep neural networks. *Journal of Biomedical Informatics* **75**, S138–S148 (2017). URL [https://www.embase.com/ search/results?subaction=viewrecord& id=L617182454&from=exporthttp: //dx.doi.org/10.1016/j.jbi.2017.06.010.](https://www.embase.com/search/results?subaction=viewrecord&id=L617182454&from=export%20http://dx.doi.org/10.1016/j.jbi.2017.06.010) <https://doi.org/10.1016/j.jbi.2017.06.010> .
7. Hassan, M. M. & Mokhtar, H. M. O. Investigating autism etiology and heterogeneity by decision tree algorithm. *Informatics in Medicine Unlocked* **16**, 100215 (2019). URL [https://www.sciencedirect. com/science/article/pii/S2352914819300541.](https://www.sciencedirect.com/science/article/pii/S2352914819300541) <https://doi.org/10.1016/j.imu.2019.100215> .
8. Leroy, G. *et al.* Automated Extraction of Diagnostic Criteria From Electronic Health Records for Autism Spectrum Disorders: Development, Evaluation, and Application. *Journal of Medical Internet Research* **20** (11), e10497 (2018). [https://doi.org/10.2196/ 10497](https://doi.org/10.2196/10497) .
9. Chen, T., Antoniou, G., Adamou, M., Tachmazidis, I. & Su, P. Automatic Diagnosis of Attention Deficit Hyperactivity Disorder Using Machine Learning. *Applied Artificial Intelligence* **35** (9), 657–669 (2021). URL [https://doi.org/ 10.1080/08839514.2021.1933761.](https://doi.org/10.1080/08839514.2021.1933761) [https:](https://doi.org/10.1080/08839514.2021.1933761)

[//doi.org/10.1080/08839514.2021.1933761,](https://doi.org/10.1080/08839514.2021.1933761)

publisher: Taylor & Francis eprint:

https://doi.org/10.1080/08839514.2021.1933761

.

1. Pruett, D. G. *et al.* Identifying developmental stuttering and associated comorbidities in electronic health records and creating a phenome risk classifier. *Journal of Fluency Disorders* **68**, 105847 (6). URL [https://ovidsp.ovid.com/ovidweb. cgi?T=JS&CSC=Y&NEWS=N&PAGE= fulltext&D=med19&AN=33894541.](https://ovidsp.ovid.com/ovidweb.cgi?T=JS&CSC=Y&NEWS=N&PAGE=fulltext&D=med19&AN=33894541) [https:](https://doi.org/10.1016/j.jfludis.2021.105847)

[//doi.org/10.1016/j.jfludis.2021.105847](https://doi.org/10.1016/j.jfludis.2021.105847) .

1. Shaw, D. M. *et al.* Phenome risk classification enables phenotypic imputation and gene discovery in developmental stuttering. *American Journal of Human Genetics* **108** (12), 2271– 2283 (2021). URL [https://www.embase. com/search/results?subaction=viewrecord& id=L2015735761&from=exporthttp: //dx.doi.org/10.1016/j.ajhg.2021.11.004.](https://www.embase.com/search/results?subaction=viewrecord&id=L2015735761&from=export%20http://dx.doi.org/10.1016/j.ajhg.2021.11.004) <https://doi.org/10.1016/j.ajhg.2021.11.004> .
2. Engelhard, M. M. *et al.* Predictive Value of Early Autism Detection Models Based on Electronic Health Record Data Collected Before Age 1 Year. *JAMA Network Open* **6** (2), e2254303 (2023). URL [https://doi. org/10.1001/jamanetworkopen.2022.54303. https://doi.org/10.1001/jamanetworkopen. 2022.54303](https://doi.org/10.1001/jamanetworkopen.2022.54303) .
3. Bishop-Fitzpatrick, L. *et al.* Using machine learning to identify patterns of lifetime health problems in decedents with autism spectrum disorder. *Autism Research: Official Journal of the International Society for Autism Research* **11** (8), 1120–1128 (2018). [https: //doi.org/10.1002/aur.1960](https://doi.org/10.1002/aur.1960) .
4. Maenner, M. J., Yeargin-Allsopp, M., Braun, K. V. N., Christensen, D. L. & Schieve, L. A. Development of a Machine Learning Algorithm for the Surveillance of Autism Spectrum Disorder. *PLOS ONE* **11** (12), e0168224 (2016). URL [https://journals.plos.org/plosone/article? id=10.1371/journal.pone.0168224.](https://journals.plos.org/plosone/article?id=10.1371/journal.pone.0168224) [https: //doi.org/10.1371/journal.pone.0168224,](https://doi.org/10.1371/journal.pone.0168224) publisher: Public Library of Science .
5. Chen, Y. H., Chen, Q. S., Kong, L. & Liu, G. D. Early detection of autism spectrum disorder in young children with machine learning using medical claims data. *BMJ HEALTH & CARE INFORMATICS* **29** (1) (2022). [https:](https://doi.org/10.1136/bmjhci-2022-100544)

[//doi.org/10.1136/bmjhci-2022-100544](https://doi.org/10.1136/bmjhci-2022-100544) .

1. Movaghar, A. *et al.* Artificial intelligence–assisted phenotype discovery of fragile X syndrome in a population-based sample. *Genetics in Medicine* **23** (7), 1273–1280 (2021). URL [https://linkinghub.elsevier. com/retrieve/pii/S1098360021050450.](https://linkinghub.elsevier.com/retrieve/pii/S1098360021050450) [https: //doi.org/10.1038/s41436-021-01144-7](https://doi.org/10.1038/s41436-021-01144-7) .
2. Shrot, S. *et al.* Prediction of tuberous sclerosis-associated neurocognitive disorders and seizures via machine learning of structural magnetic resonance imaging. *Neuroradiology* **64** (3), 611–620 (2022). URL [https:// doi.org/10.1007/s00234-021-02789-6.](https://doi.org/10.1007/s00234-021-02789-6) [https: //doi.org/10.1007/s00234-021-02789-6](https://doi.org/10.1007/s00234-021-02789-6) .
3. Morris, S. M. *et al.* Predictive Modeling for Clinical Features Associated With Neurofibromatosis Type 1. *Neurology Clinical Practice* **11** (6), 497–505 (2021). URL [https://ovidsp.ovid.com/ovidweb. cgi?T=JS&CSC=Y&NEWS=N&PAGE= fulltext&D=pmnm&AN=34987881.](https://ovidsp.ovid.com/ovidweb.cgi?T=JS&CSC=Y&NEWS=N&PAGE=fulltext&D=pmnm&AN=34987881) [https: //doi.org/10.1212/CPJ.0000000000001089](https://doi.org/10.1212/CPJ.0000000000001089) .
4. Allesøe, R. L. *et al.* Deep Learning for Cross-Diagnostic Prediction of Mental Disorder Diagnosis and Prognosis Using Danish Nationwide Register and Genetic Data. *JAMA Psychiatry* (2022). URL [https://doi.org/10. 1001/jamapsychiatry.2022.4076.](https://doi.org/10.1001/jamapsychiatry.2022.4076) [https:](https://doi.org/10.1001/jamapsychiatry.2022.4076)

[//doi.org/10.1001/jamapsychiatry.2022.4076](https://doi.org/10.1001/jamapsychiatry.2022.4076)

.

1. Alexeeff, S. E. *et al.* Medical Conditions in the First Years of Life Associated with Future Diagnosis of ASD in Children. *Journal of Autism and Developmental Disorders* **47** (7), 2067–2079 (2017). [https://doi.org/10.1007/ s10803-017-3130-4](https://doi.org/10.1007/s10803-017-3130-4) .
2. Onishchenko, D. *et al.* Reduced false positives in autism screening via digital biomarkers inferred from deep comorbidity patterns. *Science Advances* **7** (41), eabf0354 (2021). URL [https://www.science.org/doi/10.1126/ sciadv.abf0354.](https://www.science.org/doi/10.1126/sciadv.abf0354) [https://doi.org/10.1126/ sciadv.abf0354,](https://doi.org/10.1126/sciadv.abf0354) publisher: American Association for the Advancement of Science .
3. Gui, L. *et al.* Longitudinal study of neonatal brain tissue volumes in preterm infants and their ability to predict neurodevelopmental outcome. *NeuroImage* **185**, 728–741 (2019). URL [http://proxy.kib.ki.se/login?url=https: //search.ebscohost.com/login.aspx?direct= true&db=psyh&AN=2018-29772-001&site= ehost-livecristina.borradoritolsa@hcuge.ch.](http://proxy.kib.ki.se/login?url=https://search.ebscohost.com/login.aspx?direct=true&db=psyh&AN=2018-29772-001&site=ehost-live%20cristina.borradoritolsa@hcuge.ch) [https://doi.org/10.1016/j.neuroimage.2018.](https://doi.org/10.1016/j.neuroimage.2018.06.034)

[06.034](https://doi.org/10.1016/j.neuroimage.2018.06.034) .

1. SCHULER, A. *et al.* DISCOVERING PATIENT PHENOTYPES USING GENER-

ALIZED LOW RANK MODELS. *Pacific*

*Symposium on Biocomputing* **21**, 144–

(2016). URL [https://www.ncbi.nlm.nih.gov/ pmc/articles/PMC4836913/](https://www.ncbi.nlm.nih.gov/pmc/articles/PMC4836913/) .
