# Supplementary material for: Predicting neurodevelopmental disorders using machine learning models and electronic health records – status of the field
Source: J Neurodev Disord. 2024 Nov 15;16:63. doi: 10.1186/s11689-024-09579-0 (PMC11566279; doi:10.1186/s11689-024-09579-0)
Supplement: Supplementary file 2 — Supplementary Material 2. [file 11689_2024_9579_MOESM2_ESM.docx]

Key Term Box

**Area Under the Curve (AUC)**: A performance metric for classification models that measures the ability of the model to distinguish between classes. Higher AUC values indicate better model performance.

**Class Imbalance**: A situation in machine learning where the number of instances of one class significantly outnumbers the instances of other classes. This can lead to biased models that perform poorly on the minority class.

**Classical Machine Learning Methods**: These include algorithms such as logistic regression, decision trees, support vector machines, and random forests. These methods are typically used for tasks such as classification, regression, and clustering.

**Cross-Validation:** A technique for assessing the performance of a machine learning model. It involves partitioning the data into subsets, training the model on some subsets, and validating it on the remaining subsets to ensure the model generalizes well to unseen data.

**Deep Learning (DL):** A subset of machine learning involving neural networks with many layers. These networks can model complex patterns in large datasets and are used for tasks such as image and speech recognition.

**Electronic Health Records (EHRs**): Digital versions of patients' paper charts. EHRs contain patient medical history, diagnoses, medications, treatment plans, immunization dates, allergies, radiology images, and laboratory test results.

**Imputation**: The process of replacing missing data with substituted values. This technique is used to handle missing data in datasets to prevent biases and inaccuracies in machine learning models.

**Neural Networks** are a type of machine learning model inspired by the human brain's network of neurons. They consist of layers of nodes, each node being a simple mathematical model that processes inputs and passes them to the next layer. Neural networks are capable of learning features automatically avoiding hand-crafted features conventionally used in classical machine learning approaches.

**Population-Based Registers**: These are databases that collect health-related data on entire populations. They include records from various sources, such as hospitals, clinics, and government health departments.

**Sensitivity:** Also known as recall, it measures the proportion of actual positives correctly identified by the model. High sensitivity means the model correctly identifies most of the positive instances.
